
JOURNAL INFORMATION
==============================
NLM Title Abbreviation: Daniels Tex Med J
NLM Journal ID: 0006376

Daniel's Texas Medical Journal

ISSN: 0892-8487
Publisher: Biomedical Journal Digitization Project

ARTICLE INFORMATION
==============================
PMCID: PMC9176432
Article version: 1
Subjects: Book Notices

Surgical Anatomy for Students

Print publication date: 1892 Nov
Volume: 8
Issue: 5
First page: 201
Last page: 201
License: This work is free of known copyright restrictions. For more information, please see PMC Back Issue Digitization.
License URL: https://creativecommons.org/publicdomain/mark/1.0/

==============================

ries of floral diagrams; pp. 260. Price—. New York. D. Appleton & Co., 1891.

This little work is concise and practical, and certainly of much value to the busy student as he follows the more elaborate work of the professor of botany. This branch is much neglected in medical education in this country, sorry to say. B.

---

SURGICAL ANATOMY FOR STUDENTS. By A. Marmaduke Sheild, M. B. (Contab.) F. R. C. S., Senior Assistant Surgeon, Aural Surgeon, and Teacher of Operative Surgery, Charing Cross Hospital; pp. 266. Price—. New York. D. Appleton & Co., 1891.

This book is intended to be used by students in the course of preparation for their final examinations with the living model. It contains the substance of the author's demonstrations at the Charing Cross Hospital and seemed to be demanded. B.

---

A TREATISE ON BRIGHT'S DISEASE OF THE KIDNEYS. Its Pathology, Diagnosis, and Treatment, with Chapters on the Anatomy of the Kidney, Albuminuria and the Urinary Secretion. By Henry B. Millard, M. A., M. D., New York. With Numerous Original Illustrations. Third Edition, Revised and Enlarged. PP. 322. Price—. New York. William Wood & Co., 1892.

---

### Publisher's Notes.

Salophen.—At this season of the year we get our most refractory cases of rheumatism, and the old questions arise: Can we increase the doses of our remedy? Will the gastric, nephritic or cardiac functions of our patient permit a further use of the medicaments in which danger, discomfort, or perhaps failure are involved? In reviewing the combinations of salicylic acid with various bases, and especially with the phenols, the physician feels that he has here several weapons of requisite power provided the patient can withstand their use. The personal equation is the cause of trouble, and it cannot be ignored. These causes seem to have induced many able practitioners to promptly test a new
